# 1,2,4-Triazole-based anticonvulsant agents with additional ROS scavenging activity are effective in a model of pharmacoresistant epilepsy

**DOI:** 10.1080/14756366.2020.1748026

**Published:** 2020-04-07

**Authors:** Barbara Kaproń, Robert Czarnomysy, Mariusz Wysokiński, Rudolf Andrys, Kamil Musilek, Andrea Angeli, Claudiu T. Supuran, Tomasz Plech

**Affiliations:** a Department of Clinical Genetics, I Faculty of Medicine with Dentistry Division, Medical University of Lublin, Lublin, Poland; b Department of Synthesis and Technology of Drugs, Faculty of Pharmacy, Medical University of Białystok, Bialystok, Poland; c Department of Basic Nursing and Medical Teaching, Chair of Development in Nursing, Faculty of Health Sciences, Medical University of Lublin, Lublin, Poland; d Department of Chemistry, Faculty of Science, University of Hradec Kralove, Hradec Kralove, Czech Republic; e Department of Neuroscience, Psychology, Drug Research and Child Health (NEUROFARBA), Section of Pharmaceutical and Nutraceutical Sciences, University of Florence, Firenze, Italy; f Department of Pharmacology, Faculty of Health Sciences, Medical University of Lublin, Lublin, Poland

**Keywords:** 6 Hz psychomotor seizures, mitochondrial potential, total ROS activity, carbonic anhydrases, cholinesterase inhibitors

## Abstract

There are numerous studies supporting the contribution of oxidative stress to the pathogenesis of epilepsy. Prolonged oxidative stress is associated with the overexpression of ATP-binding cassette transporters, which results in antiepileptic drugs resistance. During our studies, three 1,2,4-triazole-3-thione derivatives were evaluated for the antioxidant activity and anticonvulsant effect in the 6 Hz model of pharmacoresistant epilepsy. The investigated compounds exhibited 2-3 times more potent anticonvulsant activity than valproic acid in 6 Hz test in mice, which is well-established preclinical model of pharmacoresistant epilepsy. The antioxidant/ROS scavenging activity was confirmed in both single-electron transfer-based methods (DPPH and CUPRAC) and during flow cytometric analysis of total ROS activity in U-87 MG cells. Based on the enzymatic studies on human carbonic anhydrases (CAs), acetylcholinesterase (AChE) and butyrylcholinesterase (BChE), one can assume that the herein investigated drug candidates will not impair the cognitive processes mediated by CAs and will have minimal off-target cholinergic effects.

## 1. Introduction

Epilepsy is one of the most common neurological diseases which affect around 1% of the population, including people of all ages^1^. It is characterised by recurrent episodes of seizures which result from the abnormal (i.e. excessive) electrical discharges within the nerve cells. Due to the high oxygen consumption by the brain, during these discharges huge amounts of reactive oxygen species (ROS) are produced. Since ca. 90–95% of the oxygen is metabolised during oxidative phosphorylation process, the mitochondria are the main source of ROS^2^. Much lesser amounts of ROS are, however, generated during a number of enzymatic processes that occur outside of the mitochondria (e.g. during reactions catalysed by NO synthase, COX, LOX, NADPH oxidases, xantine oxidase, etc.)^3^. There are numerous studies supporting the contribution of oxidative stress to the pathogenesis of epilepsy^4^. Importantly, oxidative stress is recognised both as the cause and the consequence of epileptic seizures^5^. The strongest evidences for a causal link between oxidative stress and epilepsy are genetic epilepsies caused by mitochondrial DNA mutations resulting in metabolic dysfunction of nerve cells^4^. Oxidative stress can lead to neuronal hyperexcitability which is considered to be an important factor in epilepsy. On the other hand, persistent epileptic seizures have been demonstrated to cause cell damage through oxidation of DNA, lipids, and proteins. ROS-induced damage to biomolecules is often observed in surgically resected samples of human brain, suggesting contribution of the oxidative stress to neuronal hyperexcitability and neurodegeneration^3^^,^^5^. Molecular markers of oxidative damage are also increased in blood samples obtained from patients suffering from temporal lobe epilepsy^3^. As it has been shown in an animal model, damage of mitochondrial DNA and excessive production of H_2_O_2_ in the inner membrane of mitochondria occur in hippocampal cells even three months after a single event of status epilepticus (SE). Therefore, the death of neurons in hippocampus is often a characteristic feature of acquired epilepsy^6^.

The abnormal concentration of the reactive oxygen/nitrogen species produced in human cells seems to be associated with the overexpression of ATP-binding cassette (ABC) transporters^7^. It results mainly from the effect of ROS/RNS on signal transduction pathways through a numerous transcription factors, e.g. NF-kB, HIF-1, Nrf2^8^. Since the main role of the ABC transporters is protecting cells from endogenous and exogenous toxins, these proteins interfere with drugs efficacy. Several studies showed that the overexpression of P-glycoprotein (P-gp), breast cancer resistance protein (BCRP), and multidrug resistance-associated proteins (MRPs) resulted in antiepileptic drugs (AEDs) resistance^7^. It is estimated that nearly 30% of epileptic patients suffer from the pharmacoresistant form of the disease^9^. Therefore, in the course of new AEDs development, there is a need for new drug candidates that combine antiepileptic effect in DRE with the ability to reduce the oxidative stress in nerve cells. As it has been observed by some authors, although many conventional AEDs (e.g. phenobarbital, phenytoin, valproate, topiramate) reduce seizure frequency, they simultaneously induce free radicals generation^4^^,^^7^. Therefore, as concluded by other authors, the use of ROS scavengers may constitute a new approach to the treatment of epilepsy.

**Figure 1.**
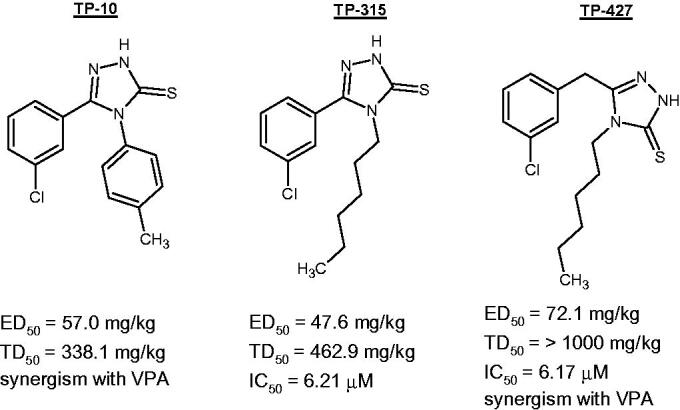
Chemical structure and pharmacological properties of the most active 1,2,4-triazole-3-thione derivatives^11^^,^^13–15^.

Our recent studies have shown that 4,5-disubstituted-1,2,4-triazole-3-thione derivatives possess promising anticonvulsant activity in animal model of generalised tonic-clonic seizures (i.e. MES test) and favourable ADME-Tox properties (long-lasting effect, low toxicity, lack of genotoxic properties, etc.)^10–16^. These compounds exhibited fast onset of action and some of them were able to potentiate the anticonvulsant activity of valproate, which is the most commonly prescribed antiepileptic drug. The use of patch-clamp and radioligand binding techniques allow us to elucidate that voltage-gated sodium channels play crucial role in the anticonvulsant activity of the above-mentioned compounds^10–12^. From the 1,2,4-triazole-3-thione derivatives investigated thus far, those with the most favourable properties (Figure 1) have been selected for further studies in order to examine their ROS scavenging activity and possible protective effect in animal model of pharmacoresistant epilepsy (6 Hz test).

Since antiepileptic agents are administered chronically to obtain seizure control, they may also contribute to the dysregulation of enzymatic systems underlying mental functions of the brain. Therefore, the last part of the research involved an investigation into the effect of compounds TP-10, TP-315, and TP-427 on human carbonic anhydrases, acetylcholinesterase, and butyrylcholinesterase.

## 2. Materials and methods

### 2.1. Investigated drugs

The following 1,2,4-triazole-3-thione derivatives were investigated during current studies: 5-(3-chlorophenyl)-4-(4-methylphenyl)-2,4-dihydro-3*H*-1,2,4-triazole-3-thione (TP-10), 5-(3-chlorophenyl)-4-hexyl-2,4-dihydro-3*H*-1,2,4-triazole-3-thione (TP-315), and 5-(3-chlorobenzyl)-4-hexyl-2,4-dihydro-3*H*-1,2,4-triazole-3-thione (TP-427). The compounds were synthesised according to procedures described in our previously published papers^13–15^.

### 2.2. Evaluation of anticonvulsant activity in 6 Hz test

All experiments on animals were performed in accordance with EU Directive 2010/63/EU for animal experiments and complied with the ARRIVE guidelines. Ethics approval for animal experimentations was obtained from the Local Ethics Committee (Lublin, Poland, approval number: 65/2018). Psychomotor seizures in mice were evoked by current (6 Hz, 0.2 ms rectangular pulse width, 32 mA, 3 s duration) generated by an S48 Square Pulse Stimulator and CCU1 Constant Current Unit (Grass Technologies, West Warwick, RI, USA). After application of ophthalmic anaesthetic (0.5% tetracaine hydrochloride) to the mice corneas, the animals underwent corneal stimulation and were placed separately in plexiglas cages (25 × 15 × 10 cm) for the observation of the presence or absence of psychomotor seizures^17^. The animals were administered with increasing doses of TP-10, TP-315, TP-427, and the anticonvulsant activity of each compound was evaluated as the ED_50_ value (median effective dose of the drug, which protects 50% of mice against convulsions) calculated using the log-probit method of Litchfield and Wilcoxon^18^.

### 2.3. Cell viability determination using MTT assay

Viability of glioblastoma cells U-87 MG (ATCC HTB-14) exposed to a fixed dose of 10 µg/ml of TP-10, TP-315, and TP-427 was measured using MTT assay. Stock solutions of the investigated compounds in DMSO were prepared in the concentration of 50 mg/ml. The cells were cultured at 37 °C in a 5% CO_2_ atmosphere using Eagle’s Minimum Essential Medium (EMEM) supplemented with 10% foetal bovine serum (FBS), 100 U/ml of penicillin, and 100 mg/ml of streptomycin. Suspension of U-87 MG cells at a density of 1 × 105 cells/ml was placed in 96-well plate (100 µl in each well) and incubated for 24 h. On the day of the experiment, the medium was removed from wells and replaced with medium containing a fixed concentration (10 µg/ml) of the respective 1,2,4-triazole derivative. After 24 h of incubation, 15 µl of MTT solution (5 mg/mL in PBS) was added to each well and the microplates were incubated for the next 3 h. Crystals of formazan formed in the metabolically active cells were solubilised with 10% SDS solution (100 µl) during overnight incubation and then the absorbance of dissolved formazan was measured at λ = 570 nm using a microplate reader (Epoch, BioTek Instruments, Inc., Winooski, VT, USA). The viability of cells treated with TP-10, TP-315, or TP-427 was expressed as the % of the viability of control cells (i.e. cells incubated without respective compounds). The experiments were run in triplicate and repeated three times. Sample observations and image capturing were performed using Olympus CKX53 microscope coupled with XM10 digital camera (Olympus).

### 2.4. Total ROS activity assay

Total ROS activity was measured using Intracellular Total ROS Activity Assay Kit (ImmunoChemistry Technologies, Bloomington, MN, USA). After 24 h of incubation of glioblastoma cells U-87 MG (ATCC, Manassas, VA, USA) with the test compounds in concentration 10 µg/ml, the medium was removed, the cells were washed twice with the cold PBS solution and the Assay Buffer (provided by the kit manufacturer) was added. Then, cells (at a density of 1 × 106 cells/ml) were treated with 10 µl of Total ROS green reagent and incubated for 1 h at 37 °C in a CO_2_ incubator. After incubation, the cells were subjected to a rinsing procedure using the Assay Buffer. Cells were suspended in 500 µl of the Assay Buffer and analysed using a flow cytometer (BD FACSCanto II flow cytometer) and FACSDiva software (both from BD Biosciences Systems, San Jose, CA, USA).

### 2.5. Flow cytometric analysis of mitochondrial membrane potential (MMP) using JC-1 dye

Mitochondrial membrane potential changes (ΔMMP) were assessed using the JC-1 MitoScreen Kit (BD Biosciences Systems, San Jose, CA, USA) as described previously^19^. Sample of 10^6^ cells/ml was suspended in a buffer mixture and incubated for 15 min at 37 °C with JC-1 dye provided by the kit manufacturer. Subsequently, the cells were washed and resuspended in a buffer. Samples prepared in this way were subjected to cytometric analysis using a cytometer BD FACSCanto II and FACSDiva software (both from BD Biosciences Systems, San Jose, CA, USA).

### 2.6. Cuprac assay

Cupric reducing antioxidant capacity (CUPRAC) assay was performed according to the method by Apak et al.^20^ with slight modifications. The CUPRAC reagent was prepared by mixing equal volume of CuCl_2_ (10 mM), neocuproine solution (7.5 mM), and acetate buffer (pH = 7.0). Subsequently, 50 µl of TP-10, TP-315, TP-427 (0.002–0.4 mg/ml) and 150 µl of CUPRAC reagent were pipetted into each well of 96-well plate (Nunc, Roskilde, Denmark). After pipetting, the plate was shaken for 5 min and then incubated for 25 min in the dark at room temperature. The absorbance of the solutions was read at 450 nm using a microplate reader (Epoch, BioTek Instruments, Inc., Winooski, VT, USA). At least six tests were performed for each concentration of the investigated compounds. The results were expressed as the IC_50_ (µg/ml) ± SD.

### 2.7. DPPH assay

Free radical scavenging activity of compounds TP-10, TP-315 and TP-427 was evaluated using DPPH assay. Briefly, 25 µl of 0.2 mM of methanolic solution of DPPH (1,1-diphenyl-1-picrylhydrazyl) was mixed with 175 µl of varying concentrations (0.002–4.5 mg/ml) of the studied compounds dissolved in methanol. Reaction mixtures in the 96-well plate were incubated in the dark at room temperature for 30 min. The absorbance was measured at 517 nm against blank (which consists of all reagents without the investigated compounds) using a microplate reader (Epoch, BioTek Instruments, Inc., Winooski, VT, USA). As a reference, the antioxidant activity of vitamin C was evaluated. Each concentration of the compounds was assayed at least six times. The results were expressed as the IC_50_ (µg/ml) ± SD calculated using the linear regression analysis.

### 2.8. Carbonic anhydrase inhibition assay

Carbonic anhydrase catalysed CO_2_ hydration activity was measured using an Applied Photophysics stopped-flow spectrometer. In brief, Phenol red (at a concentration of 0.2 mM) has been used as indicator, working at the absorbance maximum of 557 nm, with 20 mM Hepes (pH 7.5) as buffer, and 20 mM Na_2_SO_4_ (for maintaining constant the ionic strength), following the initial rates of the CA-catalysed CO_2_ hydration reaction for a period of 10–100 s. The CO_2_ concentrations ranged from 1.7 to 17 mM for the determination of the kinetic parameters and inhibition constants. For each inhibitor at least six traces of the initial 5–10% of the reaction have been used for determining the initial velocity. The uncatalyzed rates were determined in the same manner and subtracted from the total observed rates. Stock solutions of inhibitor (0.1 mM) were prepared in distilled-deionized water and dilutions up to 0.01 nM were done thereafter with the assay buffer. Inhibitor and enzyme solutions were preincubated together for 15 min or 6 h at room temperature prior to assay, in order to allow for the formation of the E-I complex. The inhibition constants were obtained by non-linear least-squares methods using PRISM 3 and the Cheng–Prusoff equation and represent the mean from at least three different determinations. All CA isoforms were recombinant ones obtained in-house^21^^,^^22^.

### 2.8. AChe and BChE inhibition assay

The inhibition ability of tested compounds against human recombinant acetylcholinesterase (hrAChE) and human recombinant butyrylcholinesterase (hrBChE) was determined by standard Ellman method adapted for 96-well plate^23^. The reaction mixture contained 10 µL of hrAChE (70 ng/mL protein final concentration) or hrBChE (220 ng/mL protein final concentration), 10 µL of tested compound (1, 10, 100, and 500 µM final concentration), 20 µL of 5,5′-dithiobis-2-nitrobenzoic acid (DTNB; 500 µM final concentration), and 60 µL of 20 mM Na-phosphate buffer (pH 7.4). The mixture was pre-incubated at 37 °C for 15 min and subsequently 10 µL substrate acetylthiocholine iodide (ATChI) or butyrylthiochiline iodide (BTChI) was added to the final concentration of 1000 µM^24^. The final volume of reaction mixture was 100 µL. The product formed during the reaction (5-thio-2-nitrobenzoic acid, TNB) was determined by measurement of its absorbance at 436 nm. The catalytic activity was evaluated as amount of product (%) formed by enzyme after 10 min of mixture incubation at 37 °C.

## 3. Results

### 3.1. Time-course and dose-response effect of TP-10, TP-315, TP-427 in the mouse 6 Hz psychomotor seizure model

All three 1,2,4-triazole derivatives possessed protective effect against 6 Hz-induced psychomotor seizures (Table 1). Peak of anticonvulsant activity was observed after 30 min (TP-10, TP-315) or 15 min (TP-427) from the *i.p.* administration of the investigated compounds. The median effective doses (ED_50_) ranged from 61.1 to 169.7 mg/kg (TP-10), 59.7 to 136.2 mg/kg (TP-315) and 40.9 to 64.9 mg/kg (TP-427). Compound TP-427 most effectively protected mice from 6 Hz-induced seizures and exerted the most beneficial time-course effect. In the cases of TP-10 and TP-315, at least two-fold decrease in ED_50_ values (as compared to the respective ED_50_ at peak activity) were observed at 60 min (ED_50_ = 169.7 mg/kg) and at 120 min (ED_50_ = 136.2 mg/kg) of the experiment, respectively. Importantly, anticonvulsant activity of TP-427 was found to be stable over the investigated time intervals. At their peak activities, all the compounds studied exhibited anticonvulsant effect from 2 to 3 times higher than valproate.

**Table 1.** Quantitative analysis of the anticonvulsant potential of compounds TP-10, TP-315 and TP-427 in the mouse 6 Hz seizure test.

Compound	Pretreatment time [min]	ED_50_ ± SEM [mg/kg]	TD_50_ ± SEM [mg/kg]	Protective index (TD_50_/ED_50_)
	15	62.6 ± 13.2	338.1 ± 12.0	5.4
TP-10	30	61.1 ± 9.7	338.1 ± 14.7	5.5
	60	169.7 ± 18.5	333.4 ± 18.6	2.0
	120	167.6 ± 17.4	395.1 ± 25.2	2.4
	15	61.3 ± 10.1	462.9 ± 20.0	7.6
TP-315	30	59.7 ± 6.8	462.9 ± 20.0	7.8
	60	68.1 ± 11.0	456.9 ± 19.7	6.7
	120	136.2 ± 18.3	448.1 ± 21.7	3.3
	15	40.9 ± 6.4	>1000	>24.4
TP-427	30	46.6 ± 8.2	>1000	>21.5
	60	51.6 ± 6.9	540.7 ± 20.9	10.5
	120	64.9 ± 5.6	548.5 ± 21.4	8.5
Valproate	15	130.6	363.3	2.8
Levetiracetam	60	19.4	>500	>25.8

Data for levetiracetam and valproate are taken from Barton et al.^25^ Median toxic doses (TD50) are taken from our previous papers^13–15^.

### 3.2. Safety profile of TP-10, TP-315, and TP-427 assessed during *in-vivo* studies

Protective indices (PI = TD_50_/ED_50_) of TP-10, TP-315, and TP-427 were evaluated using median toxic doses (TD_50_) determined in chimney test^13–15^ and median effective doses (ED_50_) from the 6 Hz-induced seizure test (Table 1). At their peak of anticonvulsant activity, compounds TP-10, TP-315, and TP-427 had PI values of 5.5, 7.8, and >24.4, respectively. All these 1,2,4-triazole-3-thiones demonstrated better safety profile than that of valproate (PI = 3.3). It is noteworthy that TP-427 exhibited PI comparable to that found for levetiracetam (PI > 25.8) which is characterised by substantially beneficial safety profile.

### 3.3. Antioxidant activity of TP-10, TP-315, and TP-427 measured in DPPH and CUPRAC assays

The investigated compounds exhibited varied antioxidant activity as measured by both 2,2-diphenyl-1-picrylhydrazyl (DPPH) and cupric reducing antioxidant capacity (CUPRAC) assays (Table 2). Ascorbic acid was used as a reference agent showing IC_50_ values of 7.82 ± 0.54 µg/ml (DPPH) and 16.05 ± 0.48 µg/ml (CUPRAC). The results from both methods were consistent and demonstrated that TP-10 had the strongest scavenging activity against DPPH radicals (IC_50_ = 31.72 ± 1.05 µg/ml) and exhibited the most potent antioxidant capacity in CUPRAC assay (IC_50_ = 16.04 ± 0.61 µg/ml). The other two compounds were less active and their IC_50_ values were in the range of 56.87–110.51 µg/ml (DPPH) and 22.28–30.92 µg/ml (CUPRAC). Interestingly, TP-10 was found to be as effective as ascorbic acid in CUPRAC assay.

**Table 2.** Antioxidant activity of TP-10, TP-315, and TP-427 determined using single-electron transfer (SET)-based methods (DPPH, CUPRAC)

	IC50 [µg/ml] ± SD	
	DPPH	CUPRAC
TP-10	31.72 ± 1.05	16.04 ± 0.61
TP-315	56.87 ± 0.70	22.28 ± 1.03
TP-427	110.51 ± 2.12	30.92 ± 1.11
Ascorbic acid	7.82 ± 0.54	16.05 ± 0.48

### 3.4. Viability of U-87 MG cells exposed to compounds TP-10, TP-315, TP-427

**Figure 2.**
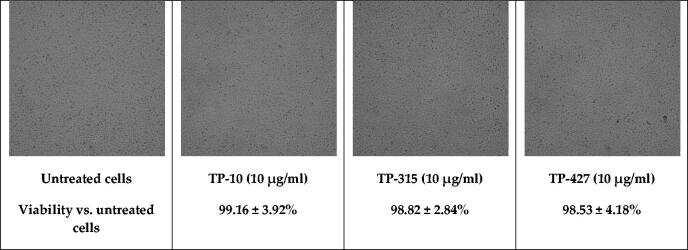
Viability of U-87 MG cells exposed to a fixed concentration of TP-10, TP-315, and TP-427.

Brain-derived U-87 MG cells were exposed to a fixed concentration (10 µg/ml) of compounds TP-10, TP-315 and TP-427. The viability of the cells was determined using MTT assay. After 24 h incubation, the investigated compounds did not significantly impair the growth of U-87 MG cells. Relative viability (%) of the cells treated with TP-10, TP-315, and TP-427 was 99.16 ± 3.92, 98.82 ± 2.84, and 98.53 ± 4.18%, respectively. Lack of toxic effects in U-87 MG cells was also confirmed on the basis of microscopic observations (Figure 2).

### 3.5. Flow cytometric analysis of total reactive oxygen species in human brain-derived cells (U-87 MG)

**Figure 3.**
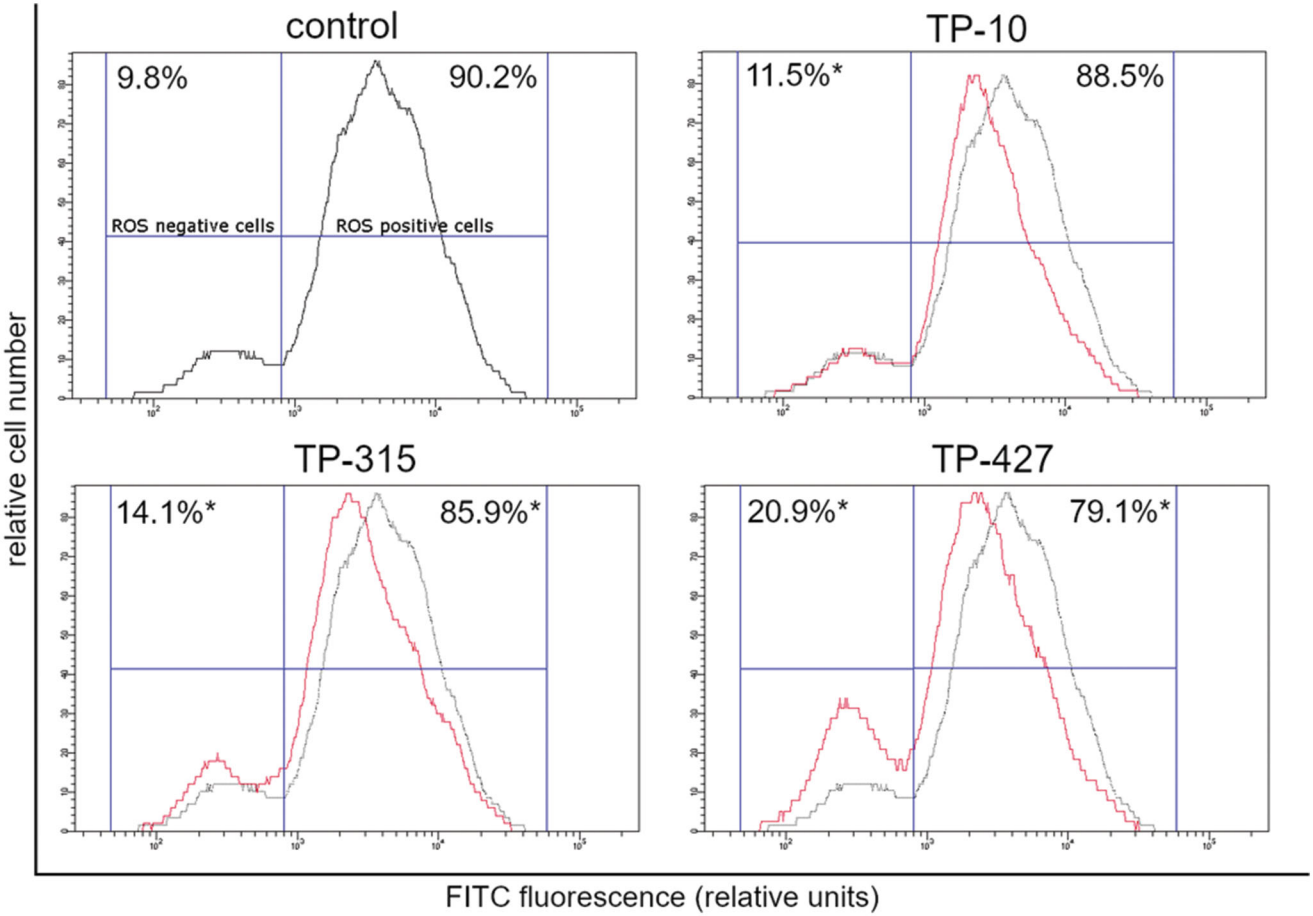
Flow cytometric analysis of U-87 MG cells treated for 24 h with TP-10, TP-315, TP-427 (red histogram) for total ROS activity. Control (untreated) cells are shown as black histogram. Mean percentage values from three independent experiments done in duplicate are presented (*p < 0.05 vs. control group).

After 24 h exposure of U-87 MG cells to TP-10, TP-315, and TP-427, the total number of ROS-positive cells decreased from 90.2% to 88.5% (TP-10), 85.9% (TP-315) and 79.1% (TP-427) (Figure 3). In the cases of compounds TP-315 and TP-427, the observed changes were designated as statistically significant (*p* < 0.05; ANOVA followed by Tukey’s multiple comparison test).

### 3.6. Flow cytometric analysis of mitochondrial membrane potential (ΔΨm) using JC-1 probe

**Figure 4.**
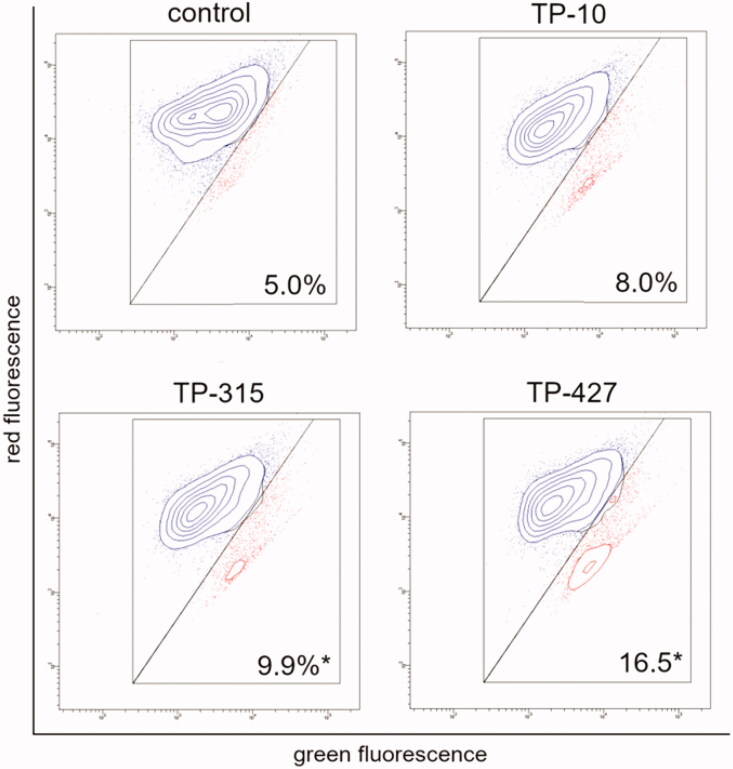
Changes of mitochondrial membrane potential of U-87 MG cells treated for 24 h with the investigated compounds (10 μg/ml). The x- and y-axes are green and red fluorescence, respectively. Mean percentage values from three independent experiments done in duplicate are presented (*p < 0.05 vs. control group).

Compounds TP-10, TP-315, and TP-427 increased the number of cells with low mitochondrial membrane potential (ΔΨm) from 5% (control cells) to 8.0%, 9.9%, and 16.5%, respectively (Figure 4). The differences in ΔΨm in control cells (untreated) and cells treated with TP-315 and TP-427 were considered as statistically significant (*p* < 0.05; ANOVA followed by Tukey’s multiple comparison test).

### 3.7. Effect of TP-10, TP-315, TP-427 on the activity of human carbonic anhydrases I and II

Inhibitory activity of TP-10, TP-315, and TP-427 against human carbonic anhydrase isoforms I and II (hCA I, hCA II) was determined using a stopped flow technique. Acetazolamide (AAZ), being a clinically used CA inhibitor, was used as a reference drug (Table 3). The investigated compounds turned out to be very weak CA inhibitors or had no inhibitory activity (K_i_ > 100 µM). Compound TP-315, containing 4-methylphenyl group at 1,2,4-triazole-3-thione core, inhibited CA I with K_i_ value of 2.19 µM. Replacement of that group with hexyl substituent in TP-315 and TP-427 resulted in a further reduction of affinity towards CA I (K_i_ = 38.78 and >100 µM, respectively). Physiologically dominant CA II isoform was not inhibited by hexyl derivatives (TP-315, TP-427) as shown by their K_i_ values (>100 µM). Compound TP-10 showed only marginal affinity (K_i_ = 74.57 µM) towards CA II after 1 h incubation, while complete lack of affinity (K_i_ > 100 µM) was observed after prolonged incubation time (i.e. 6 h).

**Table 3.** Inhibitory effect of TP-10, TP-315, and TP-427 against human carbonic anhydrases I and II.

	K_I_(µM)^a^		
	hCA I (1h)	hCA II (1h)	hCA II (6h)
TP-10	2.19	74.57	>100
TP-315	38.78	>100	>100
TP-427	>100	>100	>100
Acetazolamide	0.250	0.012	

^a^ Mean from 3 different assays, by a stopped flow technique (errors were in the range of ± 5–10% of the reported values).

### 3.8. Effect of TP-10, TP-315, TP-427 on the activity of human recombinant acetylcholinesterase (hrAChE) and butyrylcholinesterase (hrBChE)

**Figure 5.**
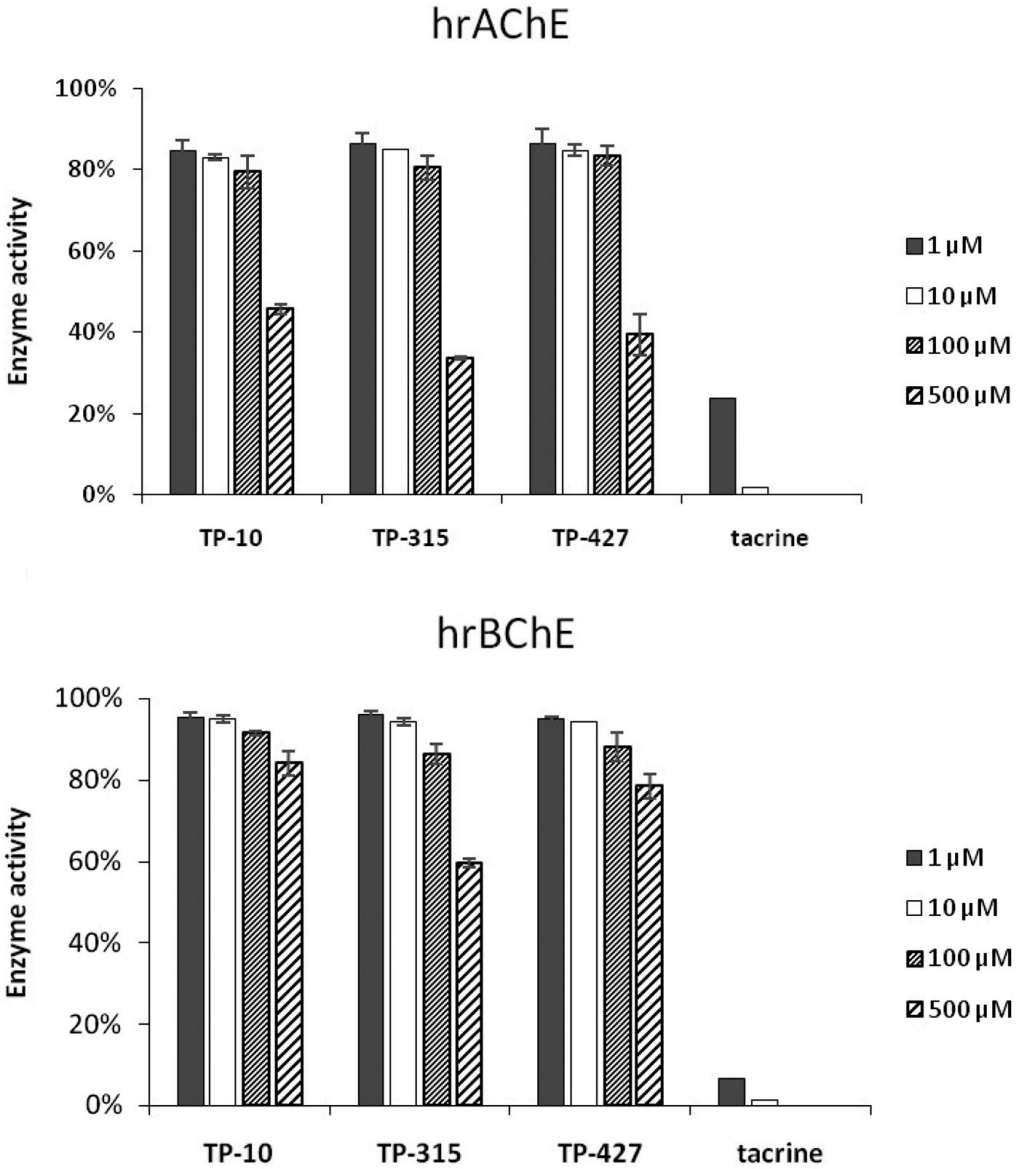
Inhibitory effect of TP-10, TP-315, TP-427 and tacrine against human recombinant acetylcholinesterase (upper graph) and butyrylcholinesterase (lower graph).

Human recombinant acetylcholinesterase (hrAChE) and butyrylcholinesterase (hrBChE) were preincubated with various concentrations (i.e. 1, 10, 100, 500 µM) of compounds TP-10, TP-315, TP-427 (Figure 5). Reference drug (tacrine) was used in concentrations of 1 and 10 µM. Only the highest concentration used in the experiments (500 µM) decreased the activity of hrAChE by ∼54% (TP-10), ∼66% (TP-315), ∼61% (TP-427). Following the exposure to lower concentrations of the tested compounds (1–100 µM), the enzyme activity remained at ∼79–85% (TP-10), ∼80–86% (TP-315), and ∼84–87% (TP-427) of the control. Butyrylcholinesterase was even less sensitive to inhibition by the investigated compounds than hrAChE. At lower concentrations (1–100 µM), hrBChE activity was still close to or higher than 90% (Figure 5, lower graph). When the hrBChE was preincubated with 500 µM of TP-10, TP-315, and TP-427, its activity decreased to 84, 59, and 78%, respectively. In summary, the investigated 1,2,4-triazole-3-thione derivatives are very poor inhibitors of hrAChE and hrBChE, suggesting minimal off-target cholinergic effects.

## 4. Discussion

Nearly 30–40% of epileptic patients exhibit low response to the pharmacological treatment. Newly developed AEDs usually offer better pharmacokinetics, milder and less frequently observed adverse effects but only slightly improved antiseizure activity^9^. Identification of brand new candidates for the treatment of pharmacoresistant form of epilepsy is mainly based on the well validated animal models^26^. One of them is 6 Hz psychomotor seizure model of partial epilepsy, originally identified in the 1950s, which is based on electrical stimulation of mice or rats with low-frequency current^25^^,^^27^. The 6 Hz test is the only acute model of drug-resistant epilepsy (the main criterion of pharmacoresistance is the resistance to at least two clinically available AEDs^28^). However, the drug responsiveness of the 6 Hz model depends highly on the current intensity. At 22 mA (equalled to CC_97_), the 6 Hz test does not discriminate between standard AEDs. But when the current intensity increases to 32 mA, the same model turns out to be insensitive to lamotrigine, phenytoin, carbamazepine and topiramate. As the current is increased to 44 mA, only a few AEDs (including levetiracetam and valproate) produce protective effects against electrically induced seizures^29^. At their peak activity, the investigated 1,2,4-triazole-3-thione derivatives appeared to have ca. 2 to 3 times more potent anticonvulsant activity than VPA in 6 Hz test in mice. Moreover, despite their activity did not achieve the level of potency observed for LEV, compound TP-427 turned out to be as safe as the mentioned AED (PI > 24.4 for TP-427 vs. PI > 25.8 for levetiracetam). Noticeably, LEV is recognised as AED with very beneficial safety profile not only in the animal studies but also in the clinical practice. Therefore, compound TP-427 fulfils very strict requirement that has been proposed for antiepileptic drug candidates.^30^

The results obtained during *in-vivo* studies indicate the influence of chemical structure of the investigated compounds on their protective activity in the 6 Hz model of pharmacoresistant seizures and acute adverse effects determined in the chimney test. Motor coordination was less impaired after i.p. administration of the respective alkyl derivatives (TP-315, TP-427) as compared with TP-10, containing 4-tolyl moiety. The two former compounds were also eliminated from the CNS more slowly than the latter one. Therefore, relatively constant anticonvulsant activity of TP-315 and TP-427 in the 6 Hz test was observed until 60 min and 120 min after their i.p. administration to mice, respectively. In turn, the constant activity of TP-10 (ED_50_ around 60 mg/kg) was noticed only within the first 30 min. Since the compounds TP-315 and TP-427 differ only by one methylene group, this structural element is undoubtedly crucial for long-lasting, strong and sustainable anticonvulsant activity in the 6 Hz model of pharmacoresistant epilepsy.

The link between oxidative stress and epilepsy has been confirmed in numerous studies on humans and animals. Different blood markers of the oxidative stress were significantly elevated in patients with epilepsy^3–5^. Even efficacious treatment with AEDs did not improve these parameters. These results were consistent with those obtained using animal models. Excessive production of ROS was observed in the kainate- and pilocarpine-treated rodents^31^. The abnormal production of hydrogen peroxide in mitochondria was found even three months after a single episode of chemically provoked status epilepticus^32^. Clinically important consequence of the elevated ROS production is the overexpression of several proteins that belong to the group of ATP-binding cassette (ABC) transporters, that contribute to AEDs pharmacoresistance.^7^ Therefore, it is desirable that new antiepileptic drug candidates combine antiseizure activity with the ability to reduce ROS generation in CNS cells. Unfortunately, many AEDs available on the market do not prevent the oxidative stress. It has been observed that phenytoin, valproate, phenobarbital and topiramate increase lipid peroxidation and elevate the level of several oxidative stress markers.

A preliminary screening of the antioxidant properties of TP-10, TP-315, and TP-427 was carried out using two methods, i.e. DPPH and CUPRAC assays. Both of them confirmed that the investigated 1,2,4-triazole derivatives exhibit free radical scavenging activity and remarkable cupric reducing power. Compound TP-10 showed the strongest antioxidant potential in both DPPH and CUPRAC assays. In the case of the latter method, TP-10 exerted activity equal to that of vitamin C. One may hypothesize that since the CUPRAC method is particularly sensitive to thiol-containing antioxidants^20^, the investigated 1,2,4-triazole-3-thiones (that can tautomerize to 1,2,4-triazole-3-thiol derivatives) were more active in this assay. However, neither DPPH nor CUPRAC methods reflect the physiological conditions in human cells. Therefore, to confirm the antioxidant potency of TP-10, TP-315, and TP-427 in *in-vivo* conditions, flow cytometric measurements of the total ROS level and mitochondrial potential were performed. Human brain-derived cells (U-87 MG) were exposed to a fixed concentration (10 µg/ml) of the compounds for 24 h. As it has been shown before in studies on TP-315 and TP-427, such a concentration remains non-toxic for human cells (HEK-293, HepG2) and is sufficient to generate the maximal anticonvulsant effect in mice subjected to the MES test^11^^,^^12^. As can be seen from Figure 2, there were also no visible changes in the morphology and viability of U-87 MG cells treated and untreated with the investigated 1,2,4-triazole-3-thiones. Exposition of the cells to the investigated compounds resulted in the decrease of the fraction of ROS-positive cells from 90.2% (control) to 88.5% (TP-10), 85.9% (TP-315) and 79.1% (TP-427). The observed changes in the intracellular ROS level were found to be associated with the loss of mitochondrial membrane potential. The number of cells with decreased ΔΨm increased from 5.0% (control) to 8.0% (TP-10), 9.9% (TP-315) and 16.5% (TP-427). Interestingly, although the antioxidant capacity of the tested 1,2,4-triazole-3-thione derivatives has been confirmed both in in-vitro and in-vivo conditions, their potency observed in DPPH and CUPRAC methods was opposite to that observed during cytometric experiments.

Importantly, 4-hexyl-5-substituted-1,2,4-triazole-3-thione derivatives (TP-315, TP-427), apart from their antioxidant activity, were also confirmed to be voltage-gated sodium channel blockers^11^^,^^12^. It is suggested by the researchers that compounds combining both antioxidant and sodium channel blocking activities are to be characterised by superior neuroprotective effect as compared to compounds possessing only one of these features^33^. For example, AM-36 – an arylalkylpiperazine agent which links these two activities, showed strong neuroprotection in *in-vitro* and *in-vivo* models of cerebral ischaemia and stroke^33–35^. Moreover, AM-36 (also known under the code name CNSB002) turned out to be an effective add-on therapy to opioids for neuropathic pain treatment^36^. Therefore, we suppose that alkyl derivatives of 1,2,4-triazole-3-thione, that combine antioxidant and VGSC blocking activities, may be of great interest not only due to their potent anticonvulsant effect but also their possible use in other disorders resulted from the extensive ROS production and excessive sodium channel activity.

In the last part of the research, the effect of the investigated compounds on the enzymatic activity of carbonic anhydrases (CA I, CA II), acetylcholinesterase (AChE) and butyrylcholinesterase (BChE) was evaluated. These enzymes are known to play important role in cognitive processes in humans. As the AEDs are often used for months, years or even for lifelong treatment, a careful consideration of the potential risks of cognitive impairments after AED candidate administration is needed. Out of 16 isoforms of CA, almost all are represented in the CNS^37^. For example, CA I is widely distributed in the motor neurons in spinal cord^38^. CA II, which is ubiquitous and physiologically dominant isoform of CA, has been identified in the myelin sheaths, myelinated tracts, oligodendrocytes, astrocytes and in the choroid plexus of mammalian brain^38^. Numerous studies, both on animal models and on humans, consistently confirm the role of CAs in cognition. Single systemic administration of acetazolamide (CA inhibitor) produced significant impairment in spatial memory, while injection of D-PHE (CA activator) to rodents resulted in the improved speed of spatial learning^39^^,^^40^. CA inhibition had also negative impact on the emotional memory acquisition and consolidation^41^. Likewise, clinical studies indicate that CAs are involved in memory and learning processes. During a randomised, double-blind, placebo-controlled clinical trial, subjects receiving acetazolamide experienced deficits in cognitive functions, attention, concentration and short-term memory^38^. Importantly, epileptic patients receiving topiramate, that apart from its anticonvulsant effect, also acts as CA I inhibitor, showed significant cognitive impairments^42^. These negative effects disappeared upon discontinuing the drug^43^. The investigated AED candidates, namely TP-10, TP-315, and TP-427, displayed very weak or no inhibitory activity against CA I and CA II. The most promising drug candidate (i.e. TP-427) completely lacked the inhibition of both CA isoforms studied, even at high micromolar concentrations. Therefore, one may assume that the compounds tested will not impair the cognitive processes mediated by CAs.

Cognitive improvements, particularly in learning and memory formation, are also observed during treatment with cholinesterase inhibitors. These drugs can delay the loss of cognitive functions and therefore are prescribed for patients with mild to moderate Alzheimer’s disease. However, cholinesterase inhibition in peripheral tissues (mainly in smooth muscles and myocardium) may result in adverse effects associated with the hyperactivity of cholinergic system. The investigated drug candidates seem not to elevate acetylcholine concentration through cholinesterase inhibition since they possess very weak inhibitory effect against hrAChE and have no inhibitory effect on hrBChE. Taking into account that main mechanism of anticonvulsant action of the compounds tested is associated with VGSC blocking, lack of AChE/BChE affinity suggests their minimal off-target cholinergic effects.

## 5. Conclusions

In this article, we showed for the first time that the investigated 1,2,4-triazole-based compounds possess anticonvulsant and antioxidant properties combined in a single molecule. At their peak activity, compounds TP-10, TP-315, and TP-427 exhibited 2 to 3 times more potent anticonvulsant activity than VPA in 6 Hz test in mice, which is well-established preclinical model of pharmacoresistant epilepsy. The antioxidant/ROS scavenging activity of the compounds tested was confirmed in both single-electron transfer (SET)-based methods (DPPH, CUPRAC) and during flow cytometric analysis of total ROS activity in U-87 MG cells. Taking into account that compounds TP-315 and TP-427 combine both antioxidant and voltage-gated sodium channel blocking activities, they may be of great interest not only due to their potent anticonvulsant effect but also their possible use in other disorders resulted from the extensive ROS production and excessive sodium channel activity. Based on the enzymatic studies on human carbonic anhydrases (CA I, CA II), acetylcholinesterase (AChE) and butyrylcholinesterase (BChE), one can assume that the herein discussed drug candidates will not impair the cognitive processes mediated by CAs and will have minimal off-target cholinergic effects.
